# Supplementary material for: A Humanized Yeast Phenomic Model of Deoxycytidine Kinase to Predict Genetic Buffering of Nucleoside Analog Cytotoxicity
Source: Genes (Basel). 2019 Sep 30;10(10):770. doi: 10.3390/genes10100770 (PMC6826991; doi:10.3390/genes10100770)
Supplement: Supplementary file 1 [file genes-10-00770-s001.zip › Additional_Files/Additional_File7_GOTermHeatmaps.zip!/Additional_File7_GOTermHeatmaps/vacuole_fusion,_non-autophagic.pdf]

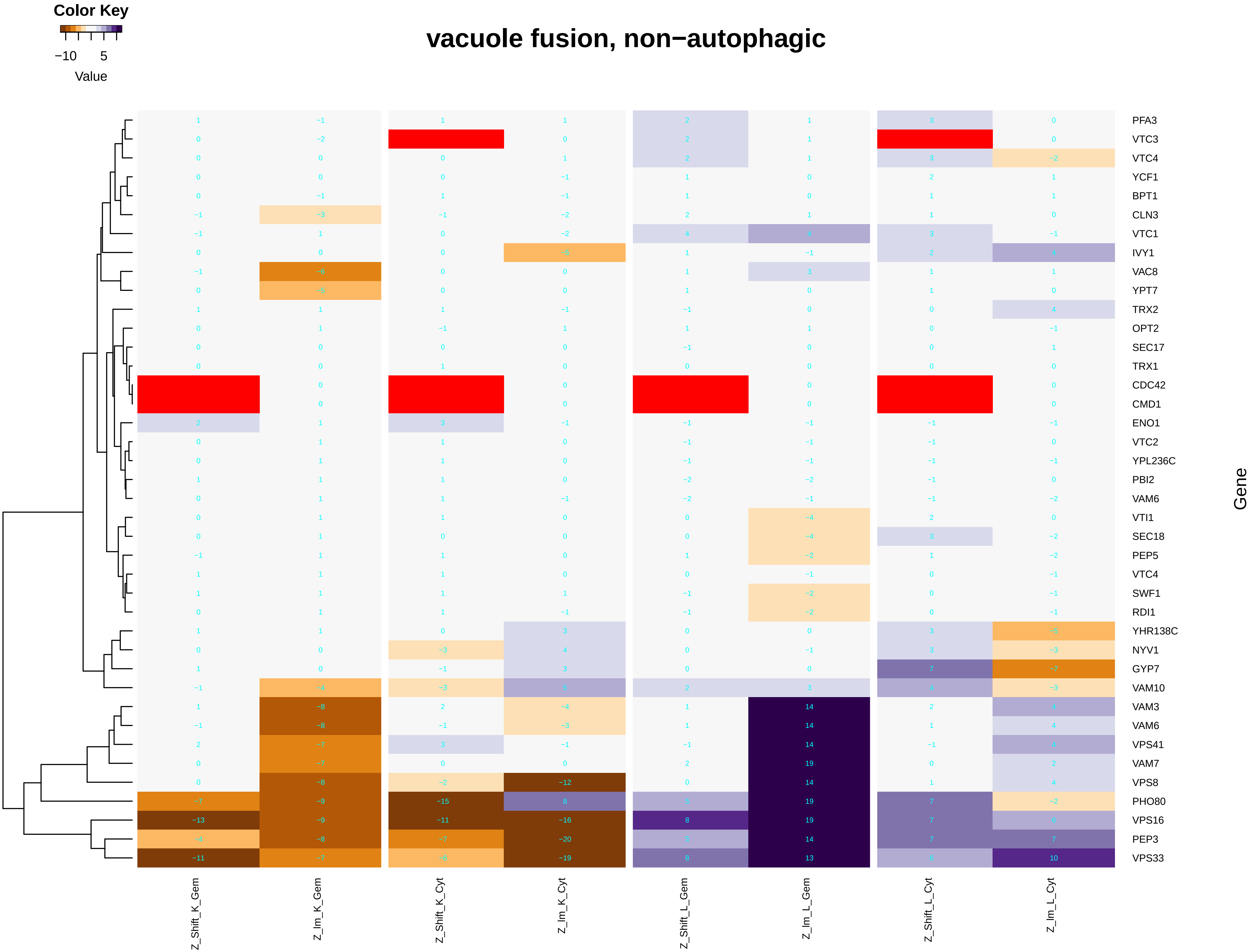

Color Key
−10
5
Value
vacuole fusion, non−autophagic
PFA3
VTC3
VTC4
YCF1
BPT1
CLN3
VTC1
IVY1
VAC8
YPT7
TRX2
OPT2
SEC17
TRX1
CDC42
CMD1
ENO1
VTC2
YPL236C
PBI2
VAM6
VTI1
SEC18
PEP5
VTC4
SWF1
RDI1
YHR138C
NYV1
GYP7
VAM10
VAM3
VAM6
VPS41
VAM7
VPS8
PHO80
VPS16
PEP3
VPS33
Gene
Z_Shift_K_Gem
Z_Im_K_Gem
Z_Shift_K_Cyt
Z_Im_K_Cyt
Z_Shift_L_Gem
Z_Im_L_Gem
Z_Shift_L_Cyt
Z_Im_L_Cyt
